# Bulk RNA sequencing dataset of Claudin-low breast cancer cell lines with Neuropilin-1 knockdown

**DOI:** 10.1038/s41597-025-06332-7

**Published:** 2025-12-01

**Authors:** Layla-Rose Lynam, Anja Rockstroh, Melanie Lehman, Yu Hin Tang, Minh Long Ngoc Nguyen, Philip A. Gregory, Colleen C. Nelson, Marianna Volpert, Brett G. Hollier

**Affiliations:** 1 Australian Prostate Cancer Research Centre - Queensland, Centre for Genomics and Personalised Health, Faculty of Health, School of Biomedical Sciences, Translational Research Institute, Queensland University of Technology, Brisbane, QLD Australia; 2 University of British Columbia, Vancouver Prostate Centre, Department of Urologic Sciences, Vancouver, BC Canada; 3 Centre for Cancer Biology, University of South Australia and SA Pathology, Adelaide, SA Australia; 4 Faculty of Health and Medical Sciences, The University of Adelaide, Adelaide, SA Australia

**Keywords:** Breast cancer, Breast cancer

## Abstract

Triple-negative breast cancers (TNBC) are a particularly aggressive breast cancer subtype with poor prognosis and high relapse rates. Due to a lack of identified targeted therapies, chemotherapy currently remains as the primary treatment for TNBC. Approximately 25–39% of TNBC are claudin-low breast cancers, which are mainly defined by low expression of cell-cell adhesion proteins and enrichment of mesenchymal signatures. Functional studies have demonstrated the potential role of the transmembrane-coreceptor, Neuropilin-1 (NRP1) in regulating the progression of these tumours. However, there have been no high-throughput studies to date that comprehensively investigate NRP1-modulated cell-signalling across multiple claudin-low cell lines. Therefore, we treated HS578T, MDA-MB-231 and SUM159PT claudin-low cell lines with either a non-targeting (NT) control or two NRP1-targeting small-interfering RNA (siRNA) or short-hairpin RNA (shRNA) sequences and followed this with bulk-RNA sequencing. We present this comprehensive transcriptomic dataset which provides a valuable resource for understanding both the transcriptomic landscape of claudin-low breast cancer and NRP1-regulated signalling pathways. Therefore, paving the way for future studies of its potential as a therapeutic target.

## Background & Summary

Breast cancer (BrCa) is a highly heterogeneous disease that can be divided into five intrinsic subtypes according to a 50-gene signature (PAM-50) classification which are luminal A, luminal B, human epidermal growth factor receptor 2 (HER2)-positive, basal-like and normal-like. Further molecular sub-classification is by estrogen receptor (ER), progesterone receptor (PR) and HER2 expression, which is defined as ER/PR-positive, HER2-enriched or triple-negative (TNBC), which do not express ER, PR or HER2^1,2^.

Triple-negative breast cancer (TNBC) comprises between 10–20% of all diagnosed breast cancers and is particularly aggressive, with metastatic disease developing in more than 30% of patients and tumour recurrence as soon as 2–3 years from diagnosis^2–7^. Because TNBCs lack expression of ER, PR and HER2, the available HER2 and hormone-receptor targeted therapies are ineffective for TNBC. This consequently results in chemotherapy remaining as the primary treatment regime^7^. Poly ADP ribose polymerase (PARP) inhibitors and Programmed cell death-ligand 1 (PD-L1) immunotherapy treatments have demonstrated significant clinical benefit, however only for subsets of TNBC that either carry BRCA mutations or are PD-L1 positive^8–10^. Hence, there is clinical demand for further research into potential targeted therapies for TNBC.

Approximately 25–39% of TNBC are further sub-classified as claudin-low breast cancer^2,11,12^. Claudin-low breast cancer was identified in 2007 and was thought to be a distinct intrinsic subtype, however research over recent years lead to a redefinition of claudin-low as an additional molecular subgroup that is acquired by the main intrinsic breast cancer subtypes^12–15^. Claudin-low breast cancer is defined by low expression of cell-cell adhesion proteins such as claudin-3, −4 −7 (*CLDN3, CLDN4, CLDN7*) and E-cadherin (*CDH1*) and enrichment of epithelial to mesenchymal transition (EMT) and cancer stem-cell signatures including Zinc finger E-box-binding homeobox 1 (*ZEB1*), Snail Family Transcriptional Repressor 1 (*SNAI1*), Vimentin (*VIM*), Integrin alpha 6 (*ITGA6*) and Aldehyde Dehydrogenase 1 (*ALDH1*)^6,12,13^. These tumours additionally exhibit aberrant activation of oncogenic signalling pathways including RAS-MAPK^6,12–15^. These characteristics result in claudin-low breast cancer being the most primitive and least differentiated subtype, with close resemblance to mammary epithelial stem cells^6,12–15^.

Neuropilin-1 (NRP1) is a 120 kDa transmembrane co-receptor protein that has been associated with several physiological processes including immunological and cardiovascular development, neuronal guidance, cell migration and angiogenesis^16^. NRP1 is known to have two main protein forms: transmembrane and soluble^16,17^. Transmembrane NRP1 is the most well characterised and consists of a small intracellular domain, transmembrane domain and an extracellular domain^18–20^. The extracellular domain function is well characterised as the binding site of most extracellular ligands and is divided into the N-terminal complement-binding CUB domain (a1/a2), coagulation factor V/VIII (b1/b2) domain, and a meprin or MAM domain (c)^16,19^. The transmembrane and meprin domains are essential for NRP1 dimerization to maintain functionality of co-receptor activity whereas the function of the intracellular domain remains unclear^18^. Soluble-NRP1 exists without the intracellular/cytoplasmic or transmembrane domains but still expresses an extracellular domain and therefore can bind extracellular ligands^16,17^. However, the functions of soluble-NRP1 are not well understood^16,17^. As a co-receptor, NRP1 dimerises to a broad spectrum of growth factor receptors including VEGFR, EGFR and PDGFR to enhance binding of the corresponding ligands^21–26^. In cancer, this consequently results in the activation of various oncogenic signalling cascades that promote tumorigenic processes such as angiogenesis, metastasis and invasion^16,27–30^.

We previously reported that NRP1 is more highly expressed in claudin-low breast cancers in comparison to Luminal A, Luminal B, HER2, Basal-like and Normal-like breast cancer^2^. Upon knockdown of NRP1 in triple-negative claudin-low cell lines, *in-vivo* tumour growth and *in-vitro* proliferation were significantly reduced^2^. Additionally, we saw decreased *ZEB1* and *ITGA6* expression as well as reduced PDGFR and EGFR activation in response to *NRP1* knockdown *in-vitro*^2^. This implicated NRP1 as a key driver of claudin-low breast cancer progression, namely through EMT and RAS-MAPK regulation^2^. However, the knowledge of the role of NRP1 in claudin-low breast cancer remains limited. Aside from a study by Al-Zeheimi *et al*.^31^ which performed transcriptional analysis of the effect of NRP1 knockout by CRISPR-Cas9 in the claudin-low MDA-MB-231 cell line, there have been no high-throughput studies to date that comprehensively investigate NRP1-modulated cell signalling across multiple claudin-low cell lines^31^.

**Fig. 1:**
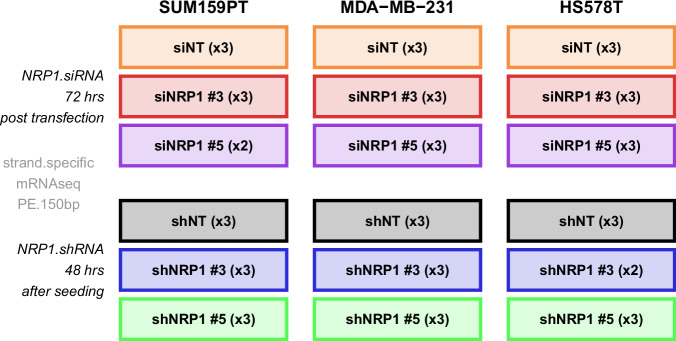
Schematic summary of dataset. Schematic summarising all cell lines and NRP1-targeting siRNA and shRNA conditions within this dataset, in addition to the number of biological replicates and experiment timepoints.

Therefore, in this study, we knocked-down *NRP1* by two small-interfering RNA (siRNA) and two short-hairpin RNA (shRNA) sequences in each of the HS578T, MDA-MB-231 and SUM159PT claudin-low cell lines^31^ and followed this with bulk-RNA sequencing. This generated a comprehensive transcriptomic dataset (consisting of 52 samples, Fig. 1) which provides a valuable resource for in depth analysis of the cellular pathways that are altered in the absence of NRP1 in these cell lines. Understanding the NRP1-regulated signalling cascades using this dataset could pave the way for future studies of NRP1 as a potential targeted therapeutic for claudin-low breast cancers, which could be administered for a substantial portion of TNBCs.

## Methods

The following sections detail the methods involved from RNA sample collection to sequencing and processing of the sequencing data. For a schematic summary of this dataset see Fig. 1. For a list and description of all 52 samples in this dataset see Tables 2–4. Two to three independent biological replicates are provided for each treatment condition.

### Cell culture

MDA-MB-231, HS578T and SUM159PT (SUM159) claudin-low breast cancer cell lines were obtained from the American Type Culture Collection (ATCC) and maintained in Dulbecco’s Modified Eagle Medium (DMEM), High Glucose, Pyruvate supplemented with 10% Fetal Bovine Serum (FBS) both from Gibco, Thermo Fisher Scientific. These cell lines were authenticated by short tandem replicate analysis at the Genomics Research Centre (Queensland University of Technology, Australia) and tested for mycoplasma using the Lonza MycoAlert mycoplasma detection kit at the Translational Research Institute (Brisbane, Australia). HEK293T cells were sourced from the ATCC and cultured in DMEM, High Glucose, Pyruvate supplemented with 5% FBS (Gibco, Thermo Fisher Scientific). All cells were maintained at 37 °C, 5% CO2.

### NRP1 knockdown by siRNA

Following the same reverse transfection methodology as previously reported^2^, 20 nM siRNA was reverse transfected into 0.7 × 10^6^ SUM159PT, MDA-MB-231 and HS578T cells using 1:500 Lipofectamine™ RNAiMAX Transfection Reagent (Invitrogen, Thermo Fisher Scientific) and 1:10 Opti-MEM™ Reduced Serum Medium (Gibco, Thermo Fisher Scientific) to DMEM supplemented with 10% FBS (Gibco, Thermo Fisher Scientific). RNA was harvested at 72 hrs post-transfection. A predesigned Silencer™ Select non-targeting siRNA (siNT) (Negative Control siRNA No. 1, #4390844, Thermo Fisher Scientific) was used in addition to two custom Silencer™ Select NRP1-targeting siRNAs (Thermo Fisher Scientific). The NRP1 siRNA sequences (sense) were as follows: siNRP1#3, 5′ UAACCACAUUUCACAAGAA 3′ and siNRP1#5, 5′ CAGCCUUGAAUGCACUUAU 3′. siNRP1#3 targets transmembrane-NRP1 only, whereas siNRP1#5 targets both transmembrane and soluble NRP1 (Fig. 6c).

### NRP1 knockdown by shRNA

Following the same shRNA transduction methodology as previously reported^2^, lentiviral media was produced by seeding 1.5 × 10^6^ HEK293T cells per shRNA in 10 mL of DMEM, High Glucose, Pyruvate supplemented with 10% heat inactivated FBS (Gibco, Thermo Fisher Scientific). FBS heat inactivation was done by a 30-minute incubation in a 56 °C water bath. At 50–60% confluency, the HEK293T cells were transfected with 200 µL of lentiviral transfection mix. Transfection mix was prepared as follows: serum-free DMEM (Gibco), 12 µL X-tremeGENE™ HP DNA Transfection Reagent (Sigma Aldrich), 1.8 µg pCMV Delta 8.2 R (Addgene), 0.2 µg pCMV-VSV-G (Addgene) and 2.0 µg of shRNA cloned to the pLKO.1 lentiviral vector (Addgene) was combined to a 200 µL final volume and incubated for 15 minutes at room temperature before adding to the HEK293T cells. Following an overnight incubation at 37 °C with 5% CO_2_, all media/transfection mix was aspirated from the HEK293T cells and replaced with 7 mL of regular culture media. Viral supernatant was then harvested at 48 hrs and 72 hrs post-transfection.

For transduction of the shRNA, 0.7 × 10^6^ HS578T, MDA-MB-231 and SUM159PT cells per shRNA sequence were cultured to 30–50% confluency. The cells were then incubated at 37 °C overnight in 4.6 mL of previously harvested lentiviral supernatant with 6 µg/mL of protamine sulfate (Sigma Aldrich). Successfully transduced cells were selected with 1 µg/mL puromycin (Gibco, Thermo Fisher Scientific) for three days before experimental use. Once 80–90% confluent post-selection, cells were seeded at 0.7 × 10^6^ and RNA was harvested after 48 hrs. Consecutive cell passages were collected as biological replicates.

The shRNAs used were a non-targeting shRNA (shNT) (Thermo Fisher Scientific), and two NRP1-targeting shRNA sequences obtained from Thermo Fisher Scientific. Sequences (sense) were as follows: shNRP1#3, 5ʹ GCUGUGGAUGACAUUAGUAUU 3ʹ and shNRP1#5, 5′ CAGCCUUGAAUGCACUUAU 3′. Similarly to the siRNA sequences, shNRP1#3 targets transmembrane-NRP1 only, whereas shNRP1#5 targets transmembrane and soluble NRP1. Note that the sequences for siNRP1#5 and shNRP1#5 are identical, whereas siNRP1#3 and shNRP1#3 sequences differ from each other, with their mapping sites being offset by about 20 nucleotides and hence, are targeting a slightly different subset of transcript variants (Fig. 6c).

### RNA Isolation, cDNA synthesis and Reverse-Transcription quantitative Polymerase-Chain-Reaction (RT-qPCR)

Cells were lysed with TRIzol reagent (Thermo Fisher Scientific, Invitrogen) and RNA extraction was performed as per manufacturer’s instructions using the Direct-zol^TM^ RNA Miniprep Plus kit (Zymo Research). RNA concentration and purity was determined using the NanoDrop™ 1000 Spectrophotometer (Thermo Fisher Scientific). A 260/280 ratio of ~2.0 was considered pure. Synthesis of 1 µg of cDNA was performed using the SensiFAST™ cDNA Synthesis Kit (Meridian Bioscience) as per manufacturer’s instructions. RT-qPCR was prepared using the SYBR™ Green PCR Master Mix (Applied Biosystems, Thermo Fisher Scientific), 10 µM of reverse and forward primers and 1:10 cDNA. The QuantStudio 6 Real-Time PCR System (Thermo Fisher Scientific) was used with the default standard run settings. Gene expression was determined using the comparative Ct method, with RPL32 as the housekeeping gene. Custom primer sequences used from Sigma Aldrich were as follows: Total NRP1 (FWD 5′ AGGACAGAGACTGCAAGTATGAC 3′, REV 5′ AACATTCAGGACCTCTCTTGA 3′, see Fig. 6c for mapping site), Transmembrane NRP1 (FWD 5′ CGAGGGCGAAATCGGAAAAGG 3′, REV 5′ CTTCGTATCCTGGCGTGCT 3′, see Fig. 6c for mapping site) and RPL32 (FWD 5′ GCACCAGTCAGACCGATATG 3′, REV 5′ ACTGGGCAGCATGTGCTTTG 3′). Statistics were determined by one-way analysis of variance (ANOVA) and Dunnett’s post-hoc multiple comparisons test within GraphPad Prism v10.0.2.

### RNA Quality validation

RNA quality and integrity was validated using the 2100 Bioanalyzer (Agilent Technologies) to determine the RNA integrity number (RIN). Sample preparation was done using the RNA 6000 Nano Kit (Agilent Technologies) according to manufacturer’s instructions. Bioanalyzer results showed that each RNA sample submitted had a RIN > 9.

### Library preparation and RNAseq

RNA was submitted to the QUT Central Analytical Research Facility (CARF), Brisbane, Queensland for library preparation and sequencing. Library preparation was performed using the Illumina TruSeq Stranded mRNA Sample Prep Kit (Illumina, strand-specific, polyA enriched) with an input of 500 ng total RNA. This was followed by paired-end sequencing using the MGI DNBSEQ-G400 sequencer with a read length of 150 bp aiming for a depth of ~40 M read pairs per sample. All samples were multiplexed across all flow cells and lanes.

### Raw data processing, alignment and quality control

For each sample, the de-multiplexed raw reads underwent quality control using the FastQC v0.11.9 tool^32^, after which the (good quality) FASTQ files from separate flow cells and/or lanes were combined, respectively. The combined FASTQ raw reads for each sample were then trimmed using TrimGalore v0.6.5^33^, followed by another quality check with FastQC. STAR aligner v2.7.2b^34^ was used for alignment to the human genome (GRCh38/hg38) and transcriptome (Ensembl.v.114/Gencode.v.48, May-2025). RSEM v1.3.3^35^ was used for read quantification, where transcript-level counts, isoform percentages and TPM values, as well as gene-level counts and TPM values were determined. Detailed tool parameters are provided in Table 1 below. Downstream data processing was performed using the R Statistical Software (version 4.4.3 2025-02-28 ucrt)^36^ scripted in the RStudio Integrated Development Environment (version 2024.12.1.563)^37^. Between-sample ‘Trimmed Mean of M-values’ (TMM) normalisation followed by counts per million (CPM) and Fragments Per Kilobase of transcript per Million mapped reads (FPKM) quantification was then completed using the R package edgeR (v.4.4.2)^38,39^. To quality control for microbial contamination, Kraken2 v2.0.9beta^40^ was used with default settings to align the unmapped reads from the STAR output to a comprehensive microbiome reference. MultiQC v1.9^41^ was used to generate STAR aligner, FastQC and Kraken2 data reports. For Multidimensional scaling (MDS) analysis, the R package edgeR (v.4.4.2)^38,39^ was used. The transcript-level and gene-level counts after TMM normalisation were used for transcript-level and gene-level MDS analysis. All plots were generated using the R package ggplot2 (v.4.0.0)^42^, as well as cowplot (v.1.2.0)^43^ to arrange composite figures.

**Table 1:** Tools, tool versions and parameters used for raw data processing.

tool version	parameters
**TrimGalore.v.0.6.5**	trim_galore --fastqc --paired --retain_unpaired --length 75 --clip_r1 10 --clip_r2 10
**STAR.v.2.7.2b**	STAR–runMode alignReads \ --readFilesCommand zcat \ --limitBAMsortRAM 80000000000 \ --quantMode TranscriptomeSAM \ --twopassMode Basic \ --alignIntronMax 1000000 \ --alignIntronMin 20 \ --alignMatesGapMax 1000000 \ --alignSJDBoverhangMin 10 \ --alignSJstitchMismatchNmax 5 -1 5 5 \ --alignSJoverhangMin 10 \ --chimJunctionOverhangMin 12 \ --chimNonchimScoreDropMin 10 \ --chimMultimapNmax 20 \ --chimMultimapScoreRange 3 \ --chimOutJunctionFormat 1 \ --chimSegmentMin 12 \ --chimScoreJunctionNonGTAG -4 \ --peOverlapMMp 0.1 \ --peOverlapNbasesMin 12 \ --outFilterMismatchNmax 999 \ --outFilterMismatchNoverReadLmax 0.04 \ --outFilterMultimapNmax 20 \ --outFilterType BySJout \ --outReadsUnmapped Fastx \ --outSAMtype BAM SortedByCoordinate \ --outWigNorm None \ --outWigType wiggle \ --outSAMunmapped Within \ --outSAMstrandField intronMotif
**RSEM.v.1.3.3**	rsem-calculate-expression --bam --paired-end --alignments --no-bam-output --seed 12345 -p 12 --forward-prob 0
**Kraken2.v2.0.9beta**	kraken2 \ --threads $task.cpus \ --db $db \ --paired \ --use-names \ --classified-out ${sample_name}-classified#.fastq \ --unclassified-out ${sample_name}-unclassified#.fastq \ --output ${sample_name}-kraken2-output.txt \ --report ${sample_name}-kraken2-report.txt \ $read1 $read2

## Data Records

This bulk mRNA-seq dataset is available for download from the NCBI Gene Expression Omnibus under GEO accession number **GSE266566**^44^ and is summarised in Fig. 1 and Tables 2–4. The GEO entry includes 104 de-multiplexed raw FASTQ files after combining lanes (R1 and R2 from paired-end sequencing), a metadata file describing the samples and the experimental details, as well as RSEM-derived transcript and gene-level raw counts, TPM values and isoform percentages. Raw data is provided on GEO as a link to the Sequence Read Archive (SRA) database.

**Table 2:** Overview of the 17 SUM159PT samples.

Sample Name	Description	GEO ID
NRP1-SUM159-siNT-RNA.1	SUM159PT with non-targeting siRNA, biological replicate 1	GSM8250668
NRP1-SUM159-siNT-RNA.2	SUM159PT with non-targeting siRNA, biological replicate 2	GSM8250669
NRP1-SUM159-siNT-RNA.3	SUM159PT with non-targeting siRNA, biological replicate 3	GSM8250670
NRP1-SUM159-siNRP1.3-RNA.1	SUM159PT with NRP1 siRNA sequence 3, biological replicate 1	GSM8250663
NRP1-SUM159-siNRP1.3-RNA.2	SUM159PT with NRP1 siRNA sequence 3, biological replicate 2	GSM8250664
NRP1-SUM159-siNRP1.3-RNA.3	SUM159PT with NRP1 siRNA sequence 3, biological replicate 3	GSM8250665
NRP1-SUM159-siNRP1.5-RNA.2	SUM159PT with NRP1 siRNA sequence 5, biological replicate 2	GSM8250666
NRP1-SUM159-siNRP1.5-RNA.3	SUM159PT with NRP1 siRNA sequence 5, biological replicate 3	GSM8250667
NRP1-SUM159-shNT-RNA.1	SUM159PT with non-targeting shRNA, biological replicate 1	GSM8250660
NRP1-SUM159-shNT-RNA.2	SUM159PT with non-targeting shRNA, biological replicate 2	GSM8250661
NRP1-SUM159-shNT-RNA.3	SUM159PT with non-targeting shRNA, biological replicate 3	GSM8250662
NRP1-SUM159-shNRP1.3-RNA.1	SUM159PT with NRP1 shRNA sequence 3, biological replicate 1	GSM8250654
NRP1-SUM159-shNRP1.3-RNA.2	SUM159PT with NRP1 shRNA sequence 3, biological replicate 2	GSM8250655
NRP1-SUM159-shNRP1.3-RNA.3	SUM159PT with NRP1 shRNA sequence 3, biological replicate 3	GSM8250656
NRP1-SUM159-shNRP1.5-RNA.1	SUM159PT with NRP1 shRNA sequence 5, biological replicate 1	GSM8250657
NRP1-SUM159-shNRP1.5-RNA.2	SUM159PT with NRP1 shRNA sequence 5, biological replicate 2	GSM8250658
NRP1-SUM159-shNRP1.5-RNA.3	SUM159PT with NRP1 shRNA sequence 5, biological replicate 3	GSM8250659

**Table 3:** Overview of the 18 MDA-MB-231 samples.

Sample Name	Description	GEO ID
NRP1-MDA.MB.231-siNT-RNA.1	MDA-MB-231 with non-targeting siRNA, biological replicate 1	GSM8250651
NRP1-MDA.MB.231-siNT-RNA.2	MDA-MB-231 with non-targeting siRNA, biological replicate 2	GSM8250652
NRP1-MDA.MB.231-siNT-RNA.3	MDA-MB-231 with non-targeting siRNA, biological replicate 3	GSM8250653
NRP1-MDA.MB.231-siNRP1.3-RNA.1	MDA-MB-231 with NRP1 siRNA sequence 3, biological replicate 1	GSM8250645
NRP1-MDA.MB.231-siNRP1.3-RNA.2	MDA-MB-231 with NRP1 siRNA sequence 3, biological replicate 2	GSM8250646
NRP1-MDA.MB.231-siNRP1.3-RNA.3	MDA-MB-231 with NRP1 siRNA sequence 3, biological replicate 3	GSM8250647
NRP1-MDA.MB.231-siNRP1.5-RNA.1	MDA-MB-231 with NRP1 siRNA sequence 5, biological replicate 1	GSM8250648
NRP1-MDA.MB.231-siNRP1.5-RNA.2	MDA-MB-231 with NRP1 siRNA sequence 5, biological replicate 2	GSM8250649
NRP1-MDA.MB.231-siNRP1.5-RNA.3	MDA-MB-231 with NRP1 siRNA sequence 5, biological replicate 3	GSM8250650
NRP1-MDA.MB.231-shNT-RNA.1	MDA-MB-231 with non-targeting shRNA, biological replicate 1	GSM8250642
NRP1-MDA.MB.231-shNT-RNA.2	MDA-MB-231 with non-targeting shRNA, biological replicate 2	GSM8250643
NRP1-MDA.MB.231-shNT-RNA.3	MDA-MB-231 with non-targeting shRNA, biological replicate 3	GSM8250644
NRP1-MDA.MB.231-shNRP1.3-RNA.1	MDA-MB-231 with NRP1 shRNA sequence 3, biological replicate 1	GSM8250636
NRP1-MDA.MB.231-shNRP1.3-RNA.2	MDA-MB-231 with NRP1 shRNA sequence 3, biological replicate 2	GSM8250637
NRP1-MDA.MB.231-shNRP1.3-RNA.3	MDA-MB-231 with NRP1 shRNA sequence 3, biological replicate 3	GSM8250638
NRP1-MDA.MB.231-shNRP1.5-RNA.1	MDA-MB-231 with NRP1 shRNA sequence 5, biological replicate 1	GSM8250639
NRP1-MDA.MB.231-shNRP1.5-RNA.2	MDA-MB-231 with NRP1 shRNA sequence 5, biological replicate 2	GSM8250640
NRP1-MDA.MB.231-shNRP1.5-RNA.3	MDA-MB-231 with NRP1 shRNA sequence 5, biological replicate 3	GSM8250641

**Table 4:** Overview of the 17 HS578T samples.

Sample Name	Description	GEO ID
NRP1-HS578T-siNT-RNA.1	HS578T with non-targeting siRNA, biological replicate 1	GSM8250633
NRP1-HS578T-siNT-RNA.2	HS578T with non-targeting siRNA, biological replicate 2	GSM8250634
NRP1-HS578T-siNT-RNA.3	HS578T with non-targeting siRNA, biological replicate 3	GSM8250635
NRP1-HS578T-siNRP1.3-RNA.1	HS578T with NRP1 siRNA sequence 3, biological replicate 1	GSM8250627
NRP1-HS578T-siNRP1.3-RNA.2	HS578T with NRP1 siRNA sequence 3, biological replicate 2	GSM8250628
NRP1-HS578T-siNRP1.3-RNA.3	HS578T with NRP1 siRNA sequence 3, biological replicate 3	GSM8250629
NRP1-HS578T-siNRP1.5-RNA.1	HS578T with NRP1 siRNA sequence 5, biological replicate 1	GSM8250630
NRP1-HS578T-siNRP1.5-RNA.2	HS578T with NRP1 siRNA sequence 5, biological replicate 2	GSM8250631
NRP1-HS578T-siNRP1.5-RNA.3	HS578T with NRP1 siRNA sequence 5, biological replicate 3	GSM8250632
NRP1-HS578T-shNT-RNA.1	HS578T with non-targeting shRNA, biological replicate 1	GSM8250624
NRP1-HS578T-shNT-RNA.2	HS578T with non-targeting shRNA, biological replicate 2	GSM8250625
NRP1-HS578T-shNT-RNA.3	HS578T with non-targeting shRNA, biological replicate 3	GSM8250626
NRP1-HS578T-shNRP1.3-RNA.1	HS578T with NRP1 shRNA sequence 3, biological replicate 1	GSM8250619
NRP1-HS578T-shNRP1.3-RNA.2	HS578T with NRP1 shRNA sequence 3, biological replicate 2	GSM8250620
NRP1-HS578T-shNRP1.5-RNA.1	HS578T with NRP1 shRNA sequence 5, biological replicate 1	GSM8250621
NRP1-HS578T-shNRP1.5-RNA.2	HS578T with NRP1 shRNA sequence 5, biological replicate 2	GSM8250622
NRP1-HS578T-shNRP1.5-RNA.3	HS578T with NRP1 shRNA sequence 5, biological replicate 3	GSM8250623

Our dataset comprises 52 samples in total, derived from the 3 claudin-low breast cancer cell lines HS578T, MDA-MB-231 and SUM159PT. For each model, NRP1 was knocked down using 2 siRNA and 2 shRNA sequences, as well as the non-targeting control RNAs, respectively. Two to three independent biological replicates (2 outlier samples were removed during data quality control) are provided for each treatment condition, resulting in 17-18 samples per cell line. See Fig. 1 for a schematic overview of the experimental setup and Tables 2–4 for a list and description of the individual samples within this claudin-low breast cancer dataset^39^.

## Technical Validation

### Validation of NRP1 knockdown by RT-qPCR

**Fig. 2:**
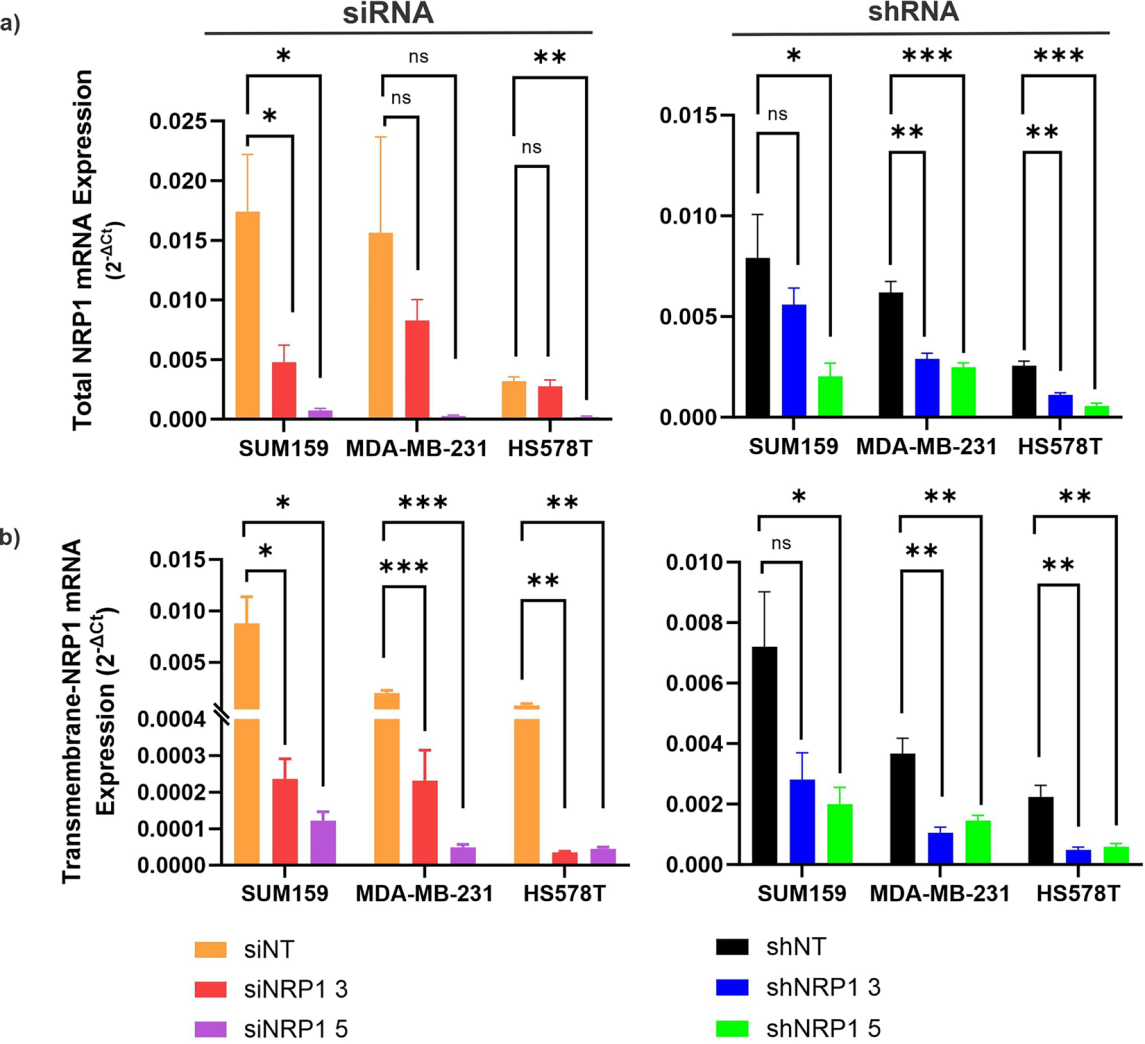
RT-qPCR validation of NRP1 knockdown in claudin-low breast cancer cell lines. mRNA expression (2^−∆Ct^) of (**a**) total NRP1 and (**b**) transmembrane-NRP1 in SUM159PT, HS578T and MDA-MB-231 cells following 72 hr transfection of siRNA (siNRP1#3 or #5) or lentiviral transduction of shRNA (shNRP1#3 and #5) and corresponding non-targeting (NT) controls, determined by RT-qPCR. Normalised to RPL32. N = 3. P-value determined by one-way analysis of variance (ANOVA) and Dunnett’s post-hoc multiple comparisons test. NS = non-significant, *p ≤ 0.05; **p ≤ 0.01; ***p ≤ 0.001. Error bars = SEM.

Before submitting for RNA sequencing, NRP1 knockdown in the RNA samples of HS578T, MDA-MB-231 and SUM159PT cells was validated by RT-qPCR (Fig. 2). RT-qPCR revealed that total NRP1 expression in the siRNA and shRNA treated samples was significantly reduced from their respective siNT or shNT controls in most cases. However, HS578T siNRP1#3 NRP1 levels were only slightly reduced and almost equal to the siNT control (fold-change of −1.15) (Fig. 2a). The siRNA and shRNA #3 sequences are identical and as they are designed to only target transmembrane-NRP1 (Fig. 6c), it was possible that soluble-NRP1 expression affected the total NRP1 knockdown levels in this sample. Therefore, we designed an additional primer set specifically for detecting transmembrane-NRP1 only (Fig. 6c). After repeating the RT-qPCR, results confirmed that transmembrane-NRP1 was repressed by shRNA and siRNA in all cell lines with statistical significance in all except SUM159PT shNRP1#3 (Fig. 2b).

### Data quality validation

**Fig. 3:**
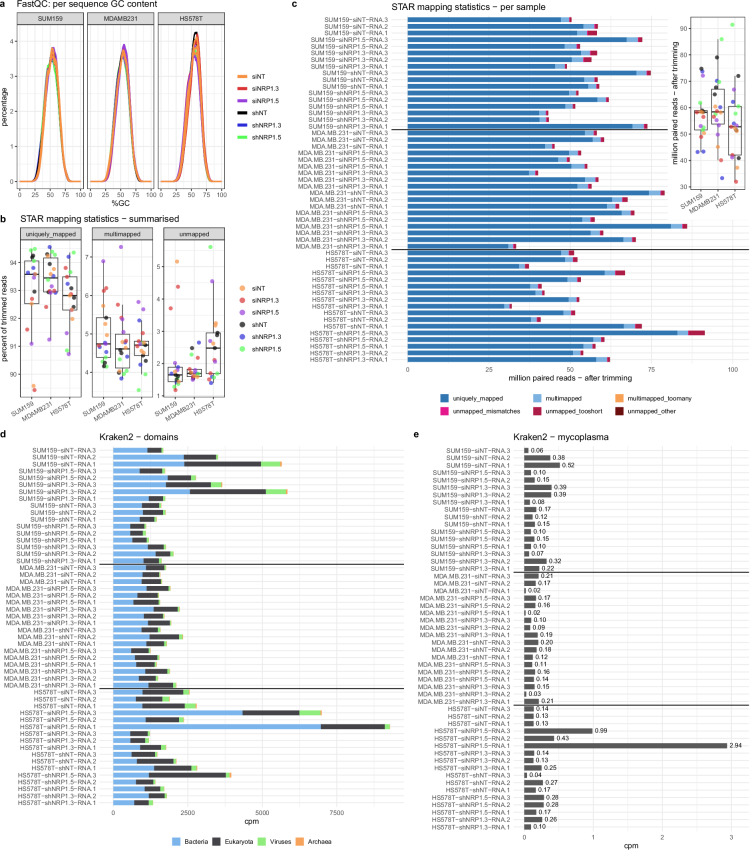
Dataset QC validation of %GC content, STAR mapping and Kraken2. Data shown is post-trimming. (**a**) Histogram of average %GC content for each sample generated by FastQC v0.11.9. (**b**) Box plot of percentage (%) of uniquely mapped, multimapped or unmapped reads to the human reference derived from the STAR aligner output. (**c**) Total STAR input reads (million paired reads) per sample with individual mapping categories as derived from the STAR aligner output. (**d**) Counts per million (cpm, calculated with respect to the total STAR input reads) for microbial domains (Bacteria, Eukaryota, Viruses, Archaea) and (**e**) mycoplasma as determined by Kraken2 v2.0.9beta. All plots were generated using the R package ggplot2 (v.4.0.0).

To analyse the quality of the sequencing data, the %GC content results generated by FastQC after trimming was first evaluated. The average %GC content per sample was plotted as a histogram and showed that all samples had a similar %GC content of ~50% (Fig. 3a).

As further validation of sequencing data quality, the number of total reads mapped to the human reference (post-trimming) as determined by the STAR aligner were analysed. Summarised as a percentage, ~85% to 95% of reads were uniquely mapped, 3%–8% were multimapped and 1% - 6% were unmapped (Fig. 3b). Altogether, there were ~30–92 M total paired reads for each sample (Fig. 3c). The mapping categories in Fig. 3c reflect the default categories provided in the STAR log files.

Additionally, the Kraken2^40^ results were analysed to screen for contamination with mycoplasma or other microbes in the reads that did not map to the human reference. Microbial domain counts per million (cpm, calculated with respect to the total STAR input reads) revealed only low-level contamination (less than 2500 cpm) with Bacteria, Eukaryota, Viruses or Archaea in most samples (Fig. 3d). Mycoplasma was detected with less than 3 cpm in all samples (Fig. 3e), confirming that the cell lines were mycoplasma free, further supporting the results obtained with the Lonza MycoAlert mycoplasma detection kit on the live cultures.

**Fig. 4:**
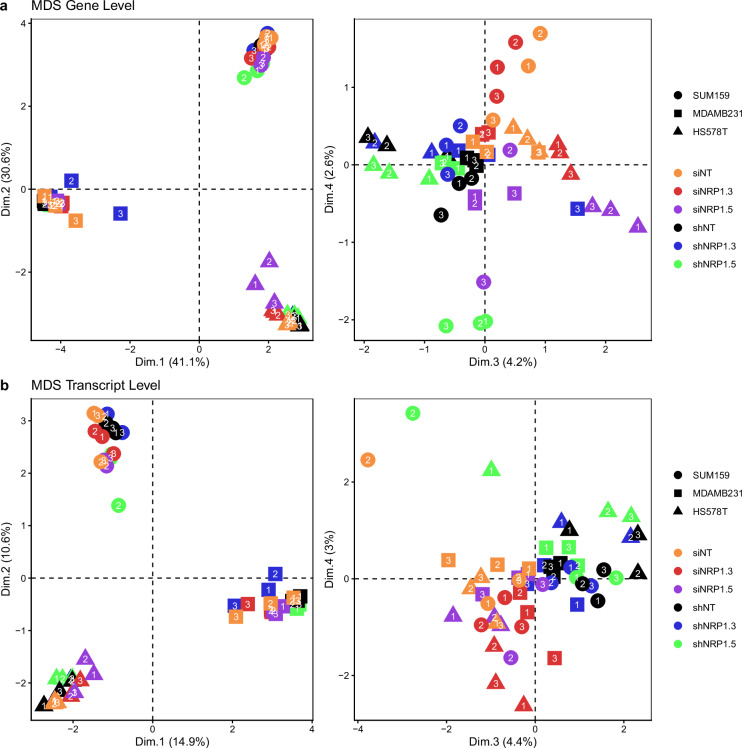
Multidimensional scaling (MDS) of all samples in the claudin-low BrCa dataset. (**a**) Gene-level and (**b**) transcript-level MDS plot based on TMM-normalised counts. Circles, squares and triangles represent SUM159PT, MDA-MB-231 and HS578T claudin-low breast cancer cell line samples, respectively. Colouring is by treatment group, with either siNT, siNRP1#3, siNRP1#5, shNT, shNRP1#3 or shNRP1#5. Data point numbers represent independent biological replicates. MDS analysis was performed using the R package edgeR (v.4.4.2) and plotted with ggplot2 (v.4.0.0).

Next, Multidimensional Scaling (MDS) was performed following TMM normalisation of the raw counts using the R package edgeR (v.4.4.2) to evaluate the similarity or dissimilarity of the samples within this dataset. Evaluating gene- as well as transcript-level MDS analyses of all samples (Fig. 4a,b) revealed that clustering was predominantly driven by cell line, indicating the large magnitude of transcriptomic differences between the models. This is followed by a separation of the samples based on the modality of knockdown (siRNA versus shRNA) in dimension 3, and a trend towards separating by target sequence #3 versus #5 in dimension 4 on the gene level.

**Fig. 5:**
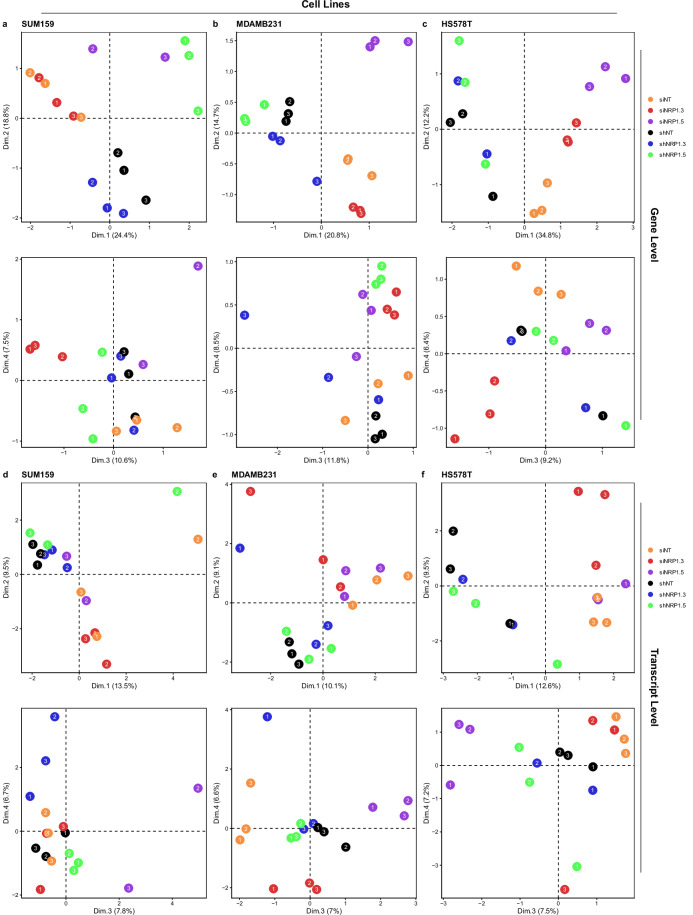
Multidimensional scaling (MDS) of the claudin-low BrCa dataset by cell line. Gene-level MDS plots for (**a**) SUM159PT, (**b**) MDA-MB-231, (**c**) HS578T as well as transcript-level MDS plots for (**d**) SUM159PT, (**e**) MDA-MB-231, (**f**) HS578T claudin-low cell lines, based on TMM-normalised counts, coloured by treatment group, with either siNT, siNRP1#3, siNRP1#5, shNT, shNRP1#3 or shNRP1#5. Data point numbers represent independent biological replicates. MDS analysis was performed using the R package edgeR (v.4.4.2) and plotted with ggplot2 (v.4.0.0).

Subsequent MDS analysis per individual cell line (Fig. 5) allowed a clearer visualisation of the treatment-group effect in each model. Although the sample clustering by type was not always tight and clean (on the transcript level in particular), gene- (Fig. 5a,b,c) and transcript-level (Fig. 5d,e,f) results showed that samples separate primarily by the siRNA versus shRNA group. In the second dimension a trend towards separation by target sequence #3 versus #5 is visible in the gene-level analysis.

**Fig. 6:**
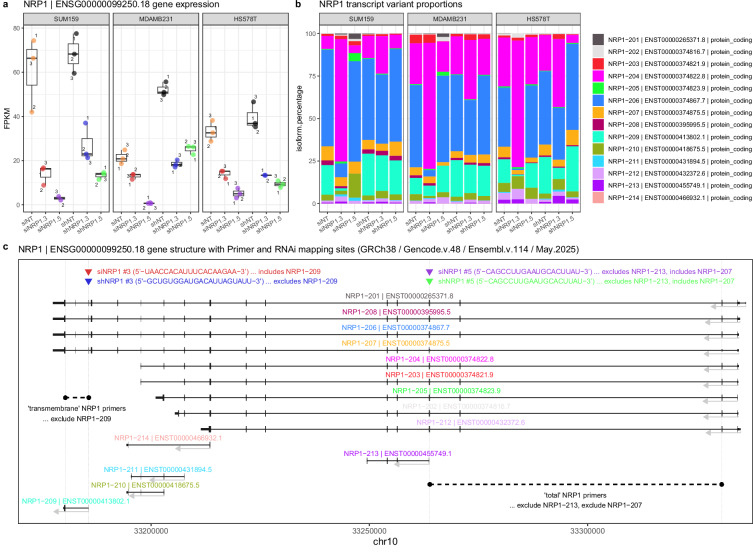
RNA-seq validation of NRP1 gene and transcript variant knockdown in claudin-low breast cancer cell lines. (**a**) Box plot of NRP1 gene expression (FPKM) and (**b**) bar chart of the isoform percentages (%) of NRP1 transcript variants in SUM159PT, MDA-MB-231 and HS578T cell lines treated with either siNT, siNRP1#3, siNRP1#5 or shNT, shNRP1#3 or shNRP1#5. (**c**) Schematic of NRP1 gene locus (as per Ensembl.v.114), showing the mapping sites of the total and transmembrane NRP1 primers, as well as the RNAi mapping sites. Note that the sequences for siNRP1#5 and shNRP1#5 are identical, whereas siNRP1#3 and shNRP1#3 sequences differ from each other, with their mapping sites being offset by about 20 nucleotides and hence, are targeting a slightly different subset of transcript variants. Plots were generated using the R packages ggplot2 (v.4.0.0) and transPlotR (v.0.0.2)^47^.

Subsequently, we evaluated NRP1 expression in our dataset at the gene and transcript level (Fig. 6). Analysis of NRP1 gene expression (Fig. 6a) revealed that in each cell line, the NRP1 expression level was lower in the siRNA and shRNA samples in comparison to their corresponding non-targeting (NT) RNAi controls, validating knockdown. Analysis of the isoform percentages of NRP1 transcript variants (Fig. 6b) showed that NRP1-206 (ENST00000374867) and NRP1-204 (ENST00000374822) had the highest expression percentage compared to other transcript variants. NRP1-206 and NRP1-204 are encoding the canonical transmembrane and soluble NRP1 isoform^45,46^, respectively (Fig. 6c). The isoform percentages further highlight that NRP1-204 (soluble variant) becomes the dominant transcript variant in the siNRP1#3 and shNRP1#3 samples, where the transmembrane-NRP1 is knocked down (Fig. 6b). This validates the specificity of the NRP1-targeting shRNA#3 and siRNA#3 sequences in knocking down only the transmembrane isoform of NRP1, and not soluble-NRP1 (Fig. 6b,c). Despite this specificity, note that the siNRP1#3 and shNRP1#3 sequences differ from each other, with their mapping sites being offset by about 20 nucleotides and hence, are targeting a slightly different subset of transcript variants (Fig. 6c). On the other hand, the sequences for siNRP1#5 and shNRP1#5 are identical, and thus, target exactly the same subset of transcript variants including transmembrane- and soluble-NRP1 isoforms.

## Usage Notes

The quality validation results demonstrated that this is a robust and reliable dataset for exploring the transcriptomic changes in response to NRP1 knockdown across multiple claudin-low breast cancer cell lines on the level of genes or individual transcript variants. Additionally, the non-targeting (NT) control RNAi samples enable the investigation and comparison of the transcriptional landscape of these claudin-low cell lines. However, it should be noted that lentiviral transduction and reverse-transfection alone can alter cellular transcriptomes. Raw data files, as well as processed/normalised data has been provided in the GEO entry so users can choose to either run their preferred raw data processing pipeline with custom parameters or utilise the processed data directly.

## Data Availability

This bulk mRNA-seq dataset with 52 samples is available from the NCBI Gene Expression Omnibus as GEO accession number GSE266566^44^. The GEO entry includes 104 de-multiplexed raw FASTQ files from paired-end mRNA-seq, a metadata file describing the samples and the experimental details, as well as RSEM-derived transcript- and gene-level raw counts, TPM values and isoform percentages. Raw data is provided on GEO as a link to the SRA database.

## Code availability

The code used to process the data and generate the figures shown in the manuscript is provided on GitHub: https://github.com/rocanja/GSE266566_NRP1_KD_BrCa_llynam.
